# Clinical Characteristics and Outcomes of Adults with Nephrotic Syndrome Due to Minimal Change Disease

**DOI:** 10.3390/jcm10163632

**Published:** 2021-08-17

**Authors:** Sophia Lionaki, Evangelos Mantios, Ioanna Tsoumbou, Smaragdi Marinaki, George Makris, George Liapis, Chrysovalantis Vergandis, Ioannis Boletis

**Affiliations:** 1 Department of Nephrology & Transplantation, Laiko Hospital, National and Kapodistrian University of Athens, 157 72 Athens, Greece; sofia.lionaki@gmail.com (S.L.); hjtdiama@yahoo.gr (I.T.); smaragdimarinaki@yahoo.com (S.M.); gkmakris@protonmail.com (G.M.); inboletis@uoa.gr (I.B.); 2 Department of Pathology, Laiko Hospital, 115 27 Athens, Greece; gliapis@gmail.com; 3 Department of Radiology, Laiko Hospital, 115 27 Athens, Greece; renaltransplant@laiko.gr

**Keywords:** nephrotic syndrome, adults, minimal change disease

## Abstract

Purpose: Minimal change disease (MCD) is considered a relatively benign glomerulopathy, as it rarely progresses to end-stage kidney disease. The aim of this study was to describe the characteristics and outcomes of adults with MCD and identify potential risk factors for relapse. Patients & Methods: We retrospectively studied a cohort of adults with biopsy-proven MCD in terms of clinical features and treatment outcomes. Baseline characteristics and outcomes were recorded and predictors of relapse were analyzed using logistic regression multivariate analysis. Results: 59 patients with adult-onset primary MCD with nephrotic syndrome were included. Mean serum creatinine at diagnosis was 0.8 mg/dL (±2.5) and estimated GFR (eGFR) was 87 mL/min/1.73 m^2^ (±29.5). Mean serum albumin was 2.5 g/dL (±0.8) and 24 h proteinuria 6.8 g (±3.7). Microscopic hematuria was detected in 35 (58.5%) patients. 42 patients received prednisone alone, six patients received prednisone plus cyclophosphamide, five patients received prednisone plus cyclosporine, one patient received prednisone plus rituximab and five patients did not receive immunosuppression at all since they achieved spontaneous remission. During a mean follow up time of 34.7(22.1) months, 46.1% of patients experienced at least one episode of relapse. The mean age of patients who did not experience a relapse was significantly higher than that of patients who relapsed while relapsers had a significantly longer duration of 24 h proteinuria prior to biopsy compared to non-relapsers. Overall, 10% of patients experienced acute kidney injury while the mean eGFR at the end was 82 mL/min/1.73 m^2^ (±29.1) and one patient ended up in chronic dialysis. Overall, the proportion of non-relapsers, who experienced acute kidney injury (17%) was significantly higher than the one recorded among relapsers (0%).Conclusion: In this series of patients, almost 46% of adult-onset nephrotic MCD patients experienced a relapse, although their renal progression was rare. Younger onset age was an independent risk factor for relapse in adult-onset MCD patients.

## 1. Introduction

Minimal change disease (MCD), which typically presents with nephrotic syndrome, is characterized by the absence of glomerular lesions by light microscopy (or only minimal mesangial prominence) and absence of staining on immunofluorescence microscopy (or low-intensity staining for C3 and IgM) while electron microscopy reveals foot process effacement with no electron-dense deposits [1,2,3,4]. More specifically, there is retraction, widening, and shortening of the foot processes [5]. It accounts for 70–90% of the nephrotic syndrome among the pediatric population younger than 10 years old and nearly 50% in older children. It is also an important cause of primary nephrotic syndrome in adolescents [4,6], with the reported rates varying between 10–15% of patients with nephrotic syndrome. The histopathologic picture of MCD is identical in adults and children. It appears that rates of MCD in adults are higher in Asia compared to Western countries [7], while the related clinical features of adults with MCD differ from those of the Western population, as it is associated with lower frequency of hypertension, microhematuria and subsequent relapses. Thus, ethnicity in addition to age is also an important predictor of the frequency of MCD among adults [8,9]. As a result, most data regarding the clinical characteristics of patients with nephrotic syndrome due to MCD come from the pediatric population or reports from Asia [7,10,11].

The aim of this study was to describe the clinical characteristics and outcomes of adults with nephrotic syndrome due to MCD treated with currently available therapies.

## 2. Materials and Methods

### 2.1. Study Design and Procedures

This is an observational study using retrospectively collected data. The participants were identified through the biopsy database of the outpatient nephrology clinic in Laiko Hospital in Athens, Greece. All patients were given a new diagnosis of MCD between January 2000 and January 2020. Inclusion criteria were age 18 years or older, biopsy-proven MCD and follow up for a minimum of six months after initiation of therapy. Demographic, clinical, and laboratory data were collected after reviewing medical charts and included information regarding the past medical history, i.e., history of arterial hypertension, diabetes mellitus, lung disease, sleep apnea, obstructive uropathy, single kidney, or malignancy, as well as smoking status and body mass index. Laboratory parameters included renal indexes, such as serum creatinine, estimated glomerular filtration rate, serum albumin, cholesterol, triglycerides, 24 h proteinuria, and hematuria. Clinical evaluation relied on the presence of edema, blood pressure measurements, serial measurements of body weight. Screening for neoplasms was performed for all patients according to the World Health Organization guidelines for each age and gender group. Recorded information also included type and duration of treatment, i.e., antihypertensive medication, diuretics, and immunosuppressants.

Protocols for immunosuppressive therapy included the following regimens: i/Oral glucocorticoids, prednisone 1 mg/kg of body weight per day, (maximum dose 80 mg/day), typically as first line therapy. Daily therapy was given as a single dose upon awakening in order to minimize adrenal suppression. The initial dose was continued for a minimum of eight weeks, even in patients who had attained complete remission earlier. In patients who achieved remission after the eight weeks prednisone tapering was initiated two weeks after complete remission was achieved. At this time the daily dose of prednisone was gradually reduced by 5 mg/day every week and once the dose reached 30 mg per day was switched to an alternate-day regimen and then tapered further over three–four months. Generally, we follow a very slow tapering in order to maintain sustained remission while the tapering may be more rapid in case of serious adverse effects. The maximum duration of daily prednisone in patients who did not attain remission was 16 weeks, while patients who did not respond by this time are considered as glucocorticoid resistant. ii/Oral cyclophosphamide 1.5–2 mg/kg of body weight per day for eight to twelve weeks combined treatment with low dose prednisone 0.3–0.5 mg/kg of body weight per day for a total of six months. iii/Cyclosporine 3–5 mg/kg of body weight per day was given in two divided doses. Serum cyclosporine levels were periodically monitored to ascertain compliance, maintenance within the therapeutic range, absorption and more importantly avoid nephrotoxic levels. Once remission was attained cyclosporine was gradually tapered and was maintained for a total of 18–24 months. In patients who failed to attain remission within six months, therapy was withdrawn. iv/Combined therapy with rituximab (375 mg/m^2^ per week for four consecutive weeks) and glucocorticoids (prednisone 0.5–1 mg/kg of body weight per day). In addition, all patients treated with glucocorticoids also received trimethoprim-sulfamethoxazole as a prophylaxis for Pneumocystis jirovecii, proton-pump inhibitors for gastroprotection and calcium complements for prevention of osteoporosis.

Outcomes of interest included remission, partial and complete, acute kidney injury, relapse, end-stage kidney disease and death from any cause. Complete remission was defined as a daily urine protein excretion of <0.3 g/d, while partial remission was defined as >50% reduction in proteinuria from baseline, which remained below the nephrotic range. Acute kidney injury (AKI) was defined as an acute rise in serum creatinine of ≥50%, either known or presumed to have occurred in the preceding seven days. Relapse was characterized by recurrence of proteinuria in the nephrotic range or higher in patients who had previously undergone a complete or partial remission. Patients were considered frequent relapsers if they had three or more relapses per year. Glucocorticoid dependence was considered in patients who experienced a relapse while on therapy or required continuation of steroids to maintain remission. Glucocorticoid resistance was considered in patients with little or no reduction in proteinuria after 16 weeks of therapy with glucocorticoids, or in rare cases, who had some reduction in proteinuria with more prolonged therapy, but never met the criteria for partial remission.Patients were grouped on the basis of experiencing a relapse or not in order to identify risk factors for relapse among patients with MCD. Only patients who continued to be followed in the related clinic of our department were includedin the multivariate ordinal logistic regression analysis for identification of risk factors for relapse after achievement of remission with periodical visits usually every three months. The estimated glomerular filtration rate (eGFR) was calculated using the four-variable Chronic Kidney Disease Epidemiology Collaboration (CKD-EPI) study equation [11]. Proteinuria was measured using 24 h urine collection. Urine analysis wasperformed every two weeks during the initial time after diagnosis and every three months afterwards in patients who were in stable clinical condition follow up responding to immunosuppressive therapy or after achievement of remission. A related sentence has now been added in the text. Dip stick for more frequent check of albuminuria was not used.

### 2.2. Statistical Methods

Available case analysis was performed for univariate and bivariate exploratory analyses. Categorical variables were expressed as a number and percentage, and continuous variables are expressed as a mean and standard deviation (SD). Differences between continuous variables were tested for statistical significance using Student’s *t*-test. Due to sample size considerations, exact methods were used for the analysis of categorical variables (Fisher’s exact test or an exact chi-square test). Statistical significance was taken to be a *p* value < or equal to 0.05. Factors associated with the occurrence of one or more relapse episodes were also examined by univariate and multivariate ordinal logistic regression analyses. The variables were chosen based on literature reports and adjusting for possible confounding factors. Odds ratios and 95% confidence intervals are reported to show the magnitude and significance of associations. The Stata 13.1 Statistical Software package (StataCorp, College Station, TX, USA) was used in statistical analyses.

## 3. Results

### 3.1. Description of Study Population

Fifty-nine patients with biopsy-proven MCD who were diagnosed between January 2000 and January 2020 were studied retrospectively. Demographic, clinical, and laboratory data at the time of renal biopsy are presented in Table 1. The mean age at the time of MCD diagnosis was 47 (±17) years, while 59.3% of patients were males. Among females, 41.6% were in menopause. Ninety eight percent (98%) of patients were Caucasians. Concerning past medical history 38.9% of patients had already been diagnosed with hypertension at presentation and two of them (3.3%) had been diagnosed with diabetes mellitus. At the time of renal biopsy, glomerular filtration rate was within the normal range in the vast majority of patients. Mean serum creatinine was 0.8 mg/dL (±2.5) and mean eGFR was 87 mL/min/1.73 m^2^ (±29.5). Mean serum albumin was 2.5 g/dL (±0.8), mean cholesterol level was 360.5 mg/dL (±118.15) and mean urinary protein excretion was 6.8 g/day (±3.7). Microscopic hematuria was detected in 35 (60%) patients. Six patients experienced acute kidney injury while the mean eGFR at end was 82 mL/min/1.73 m^2^ (±29.1). Five out of six recovered and one progressed to end stage CKD. All six patients suffered from AKI at initial presentation or one month after initial presentation. Five patients did not receive immunosuppressive treatment since they achieved complete spontaneous remission without experiencing a subsequent relapse. Initial immunosuppressive therapy consisted of prednisone alone in 42 cases (77.7%), cyclophosphamide plus prednisone in six (11.1%) cases, cyclosporine plus prednisone in five cases (9.2%) and rituximab plus prednisone in one case. As far as the side effects of corticoid therapy are concerned, three patients developed diabetes mellitus and two suffered from serious infections during steroid treatment. These two patients suffered from bacteremia due to urinary tract infection and recovered with antibiotic therapy. Furthermore, one patient developed osteoporosis four months after the beginning of steroid tapering.

**Figure 1:**
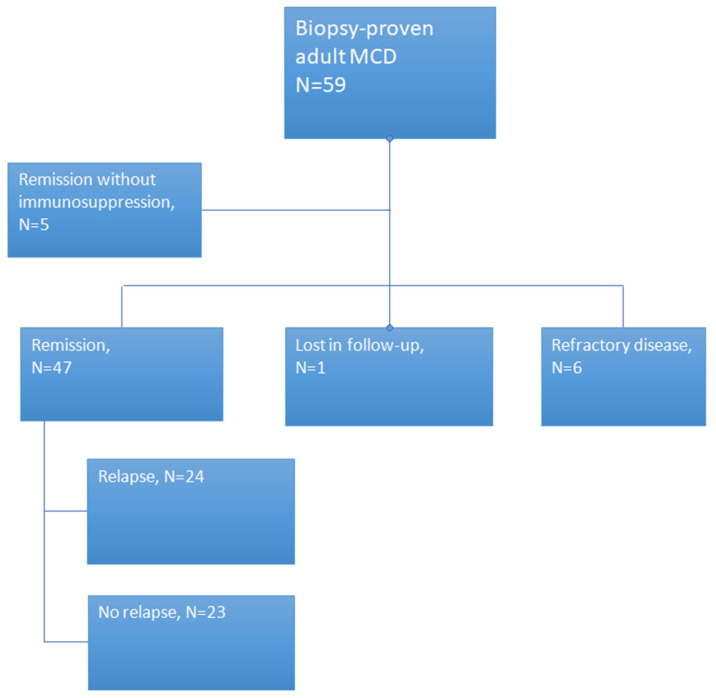
Study design.

Overall, 52 out of 59 patients (88.1%) achieved remission. The mean time to remission was 41 days from initiation of therapy. Among 59 patients with biopsy-proven MCD, six (10.1%) patients never achieved remission, five (8.4%) achieved complete remission without any immunosuppressive therapy (spontaneous remission), and one (1.6%) patient had inadequate follow-up data and thus he was excluded from further analysis. Of the 47 patients who achieved complete or partial remission with immunosuppressive therapy, there were 24 patients (51.06%) who experienced at least one episode of relapse, and 23 (48.9%) who were non-relapsers. Six patients (10.1%) manifested glucocorticoids resistance after initial therapy (Figure 1). Norepeat biopsy was done to patients with steroid resistance, since one of them died due to sudden cardiac arrest, three of them had not completed 16 weeks of therapy when data was collected and two of them initiated prednisone plus cyclosporine after 16 weeks of prednisone monotherapy as possible FSGS patients without biopsy due to technical difficulties and complications in their biopsy.

#### 3.1.1. Relapsing Patients

At least one episode of relapse occurred in 24 (46.1%) patients of those who achieved remission (after initial therapy, immunosupressive or not) during a mean observation period of 34.7 months. Most of the relapses occurred in the first six months of therapy, soon after remission and during prednisone tapering. Nearly 17% of patients experienced one episode of relapse, 13% of patients experienced two episodes of relapse, and 15% of patients experienced relapse three times during the observation period. Among patients who received combined therapy with glucocorticoids and cyclosporine, two patients did not respond and three patients achieved complete remission, while one patient that achieved remission relapsed later. All of the patients who received glucocorticoids and cyclophosphamide attained complete sustained remission but three of them experienced subsequent relapse. During the 2nd or 3rd episode of relapse the patients received the following therapies; all patients were treated with glucocorticoids, in 10 cases glucocorticoid was given as monotherapy, while in 13 cases it was given in combination with cyclophosphamide (8 patients, 34.7%), or calcineurin inhibitors (5 patients, 21.7%) Twenty-two of 23 cases that relapsed for second or third time achieved remission with these therapies.

#### 3.1.2. Relapsers versus Non-Relapsers

A comparison of the demographics and laboratory characteristics between patients who experienced at least one episode of relapse and those who didnot is displayed in Table 2 and Table 3. The mean age of patients who did not experience a relapse after achievement of remission was significantly higher than that of patients who relapsed. Relapsers had a significantly longer duration of proteinuria prior to biopsy compared to non-relapsers (*p* = 0.05). Table 4 shows the comparison of treatment and outcomes between relapsers and non-relapsers. At the end of the observation period the mean eGFR was not different between groups (85.1(±27.8) versus 92.6(±23.9) mL/min/1.73 m^2^). Interestingly, the proportion of non-relapsers who experienced AKI during the observation period (17%) was significantly higher than the one recorded among relapsers (0%) (*p* = 0.04). By multivariate logistic regression analysis, it was shown that the risk of experiencing a relapse episode was reduced by 9% for every yearly increase in the age of disease onset (odds ratio [OR], 0.92; 95% confidence interval [CI], 0.83–0.99; *p* = 0.035), adjusted for follow-up time (Table 5).

**Figure 2:**
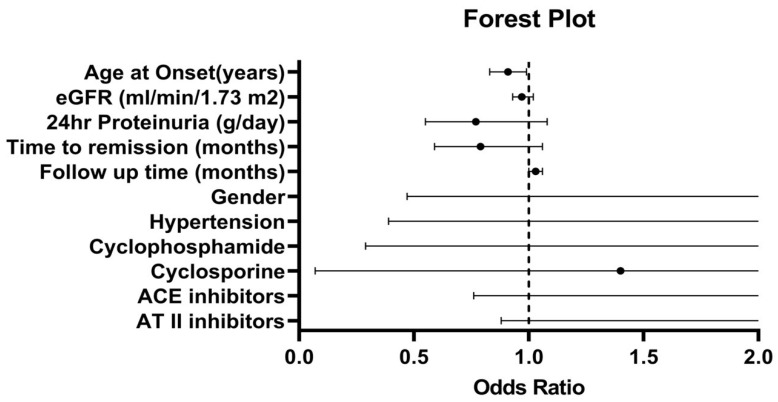
Forest plot based on the results of multivariate analysis of patients with MCD who experienced a relapse and those who did not.

**Figure 3:**
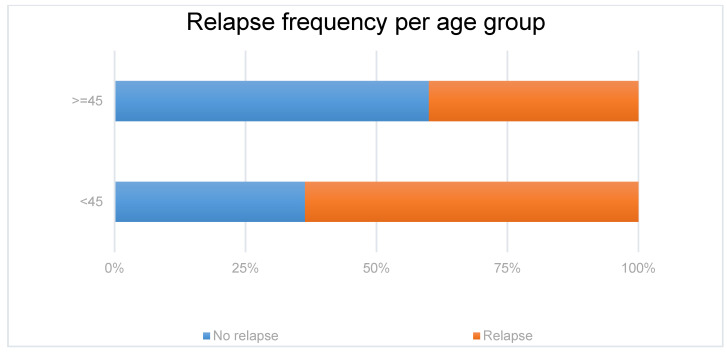
Relapse frequency of patients with MCD depending on the age group to which they belonged (older or younger than 45 years old).

The forest plot (Figure 2) shows that only age at onset was proved to be a strong independent risk factor for a relapse. Forty per cent of patients more than 45 years old experienced a relapse, while 65% of patients less than 45 years of age experienced a relapse (Figure 3).

## 4. Discussion

This retrospective investigation studied the clinical characteristics and treatment outcomes of adults Caucasians with nephrotic syndrome due to biopsy-proven MCD. In this cohort of patients with MCD, the mean age at diagnosis was 47 (±17) years and the majority of patients were males. The most frequent clinical manifestations were weight gain and edema, which were the reasons for seeking medical attention in most cases. Renal function was within the normal range and mean 24 h proteinuria was 6.8 g/day (±3.7). Microscopic hematuria was detected in 60% of patients and most patients were treated with glucocorticoids alone as first line therapy. Microscopic hematuria is generally common in adults with MCD and occurs in approximately 25% of children although the clinical course is not different [12,13]. In this cohort, 10% of patients developed AKI during the follow up time, which was associated in most cases with hemodynamic changes due to nephrotic syndrome and hypoalbuminemia. Generally, serum creatinine concentration in patients with MCD has been reported to be modestly elevated at presentation in both adults and children [14]. Waldman et reported that among 95 adults with MCD, 25.2% experienced AKI, which was associated with older age, hypertension, and hypoalbuminemia [12]. However, Fenton et al. reported that 37% were in AKI at presentation, which was significantly associated with a lower serum albumin and older age [15].

The risk of relapse was reduced by 9% for every year of increase in the age of disease onset. Likewise, in previous reports relapse was observed in 54% of adult patients who were initial responders, while comparison between the relapsing and non-relapsing patients revealed only proteinuria at diagnosis to be significantly different. In the same study proteinuria greater than 7 g/day at presentation was associated with subsequent relapse [16]. Waldman et al. found that relapse of nephrotic syndrome occurred in 73.1% of initial responders with no significant differences in the relapse rate between daily and alternate-day treatment with glucocorticoids [12]. Approximately 76% of included patients were treated with at least one additional course of steroids and 91.70% achieved remission, with a complete remission rate of 84.40% [12]. Other reports of adults with MCD found that 30–62% of patients experienced a single relapse episode and up to 39% had frequent relapses [17,18].

The fact that younger age onset is a risk factor for relapse in patients with minimal change disease is confirmed by data from child cohorts. Ruggenenti et al. recorded the efficacy of rituximab in 30 patients with steroid-dependent or frequently relapsing idiopathic nephritic syndrome, 20 of whom were adults and 10 of whom were children. Younger age at inclusion significantly correlated with a higher number of relapses before rituximab administration [19]. Trautmann et al. also confirmed through a multivariate analysis that age betweenone and six years is a risk factor for the development of ESRD in children with steroid resistant nephrotic syndrome, probably due to the higher number of relapses in these patients [20].

Yet, recently, a larger study from Japan, which included 192 adults with MCD where the researchers aimed to assess the association between steroid dose used for relapse and subsequent outcomes, 96.9% achieved complete remission and 52.1% relapsed subsequently [21]. They also found that, during a median observation period of 37.6 months, clinical outcomes of patients with MCD, who were treated with a lower dose of glucocorticoids (10–20 mg/day), did not differ from those who were treated with higher doses (>20 mg/day). Thus, they concluded that a higher dose of glucocorticoids ma30y not be necessary for the treatment of adult MCD relapse [21]. Fenton et al. [15] reported that 98% of 78 patients with MCD and a mean age of 36 years achieved remission at a median time of five weeks, while 61% relapsed. A higher eGFR was associated with an increased risk of relapse, and females were significantly more likely than males to have an early relapse. In general, longstanding experience has shown that in adults, relapses are frequent, occurring in about 56–76% of patients [12,22,23]. Importantly, in patients with MCD, the occurrence of chronic renal failure is exceptional, probably related to the fact that the disease is generally responsive to glucocorticoids. Patients who do not experience any relapses within two years from treatment discontinuation are considered to have attained sustained remission [24,25]. Infrequent patients may relapse years after they have been declared cured. Long-term outcome studies of large cohorts of childhood onset MCD followed into adulthood show about 3% developing chronic renal failure and 16–42% recurring in adulthood. In this population, the main risk factor for adult relapses is frequent relapses in childhood [24,25]. Nevertheless, morbidity associated with the nephrotic state itself and the immunosuppressive treatment of MCD remains problematic [25,26,27]. The issue of cumulative toxicity is even more remarkable among individuals with childhood-onset MCD, who experience disease relapses or steroid-dependent nephrotic syndrome during adulthood. This population, as well as patients with adult onset of MCD with multiple relapses, should be followed closely for sterility associated with cytotoxic agents, nephrotoxicity with calcineurin inhibitors, diabetes, hypertension, obesity, osteoporosis, cataracts related to steroids, and solid or blood neoplasia because of prolonged immunosuppression [26,27,28]. Rituximab, a monoclonal antibody against CD 20 that has been used for the treatment of MCD in both children and adults, was used in one of the patients in our series. Observational studies suggest that this agent may be efficacious in adults with frequently relapsing or glucocorticoid-dependent MCD [29,30,31,32]. Furthermore, rituximab may be attempted in patients who have failed to achieve remission withcyclophosphamide or calcineurin inhibitors. By contrast, rituximab does not appear to be effective in adults with glucocorticoid-resistant MCD and therefore should be avoided in these patients. A study that evaluated the efficacy and safety of rituximab in 17 adults with glucocorticoid-dependent MCD over a mean follow-up of 29.5 months found that remission was achieved in 11/17 patients while 9/11 were able to discontinue all other immunosuppressants [29]. Among these, there were six patients who relapsed within a mean time to relapse of 11.9 months. No adverse events were recorded. Potentially, rituximab represents the most promising therapy in patients with multiple relapses due to MCD, since it has been associated with a reduction in the median number of relapses and weaning of immunosuppressive therapy [33].

Limitations to this study pertain to it retrospective design, while it is largely a referral-based cohort. Therefore, it may not be representative of all adult patients with MCD. Since the data were retrospectively collected, they are dependent upon the accuracy and completeness of hospital databases. The data were neither created nor collected to answer a specific hypothesis but have been used to provide an outline of the adult MCD cohort in our center.

In conclusion, we describe here a cohort of patients with biopsy-proven MCD. Of these, almost 46% experienced at least one episode of relapse following remission, although their renal progression was rare. Younger onset age was shown to be an independent risk factor for relapse in adult-onset MCD patients.

## Figures and Tables

**Table 1:** Demographics, clinical features and treatments outcomes of patients with MCD in adulthood.

Characteristic	Mean (SD) or *N* (%)
Age at start of symptoms	47 (17.25)
Proteinuria duration before biopsy (weeks)	8.8 (22.5)
Age at diagnosis (biopsy)	47 (17.25)
Gender (males)	35 (59.3)
Menopause (yes)	10 (41.6)
Race (Caucasians)	55 (98.2)
History of Smoking status	14 (23.7)
Current	8 (57.1)
Ex-smoker	2 (14.3)
Never	4 (28.6)
BMI	26.9 (4.7)
Serum creatinine at biopsy, (mg/dL)	0.8 (2.5)
Serum total protein, (g/dL)	4.8 (1.1)
Serum albumin, (g/dL)	2.5 (0.8)
Serum cholesterol, (mg/dL)	360.5 (118.15)
Triglycerides, (mg/dL)	177.5 (87.5)
Estimated GFR mL/min/1.73 m^2^	87 (29.5)
24 h proteinuria biopsy (g)	6.8 (3.7)
Urine analysis protein at biopsy	
+	0
++	9 (15.2)
+++	27 (45.7)
>3+	22 (37.2)
Urine analysis hematuria at biopsy	58
+	24 (40)
++	8 (13.5)
+++	3 (5)
>3+	0
Edema at biopsy	
+	11 (18.6)
++	16 (27.1)
+++	9 (15.2)
>3+	12 (20.3)
History of Diabetes mellitus	2 (3.3)
History of hypertension	23(38.9)
History of Lung disease	3(5)
Single kidney	1(1.6)
Malignancy	3 (5)
**Treatment for MCD**	
ACE Inhibitor,	26 (55.9)
AT-II (yes)	6 (10)
Diuretics	29 (49.15)
Other anti-hypertensives	20 (33.8)
Glucocorticoids alone	42 (77.7)
Cyclosporine plus glucocorticoids	5 (9.2)
Cyclophosphamide plus glucocorticoids	6 (11.1)
Rituximab plus glucocorticoids	1(1.8)
Patients with Remission (complete or partial)	52/59 (88.1)
Patients with Relapse	24/52 (46.1)
Relapse (episodes per patient)	2.4 (2.1)
Acute kidney injury	6 (10)
Acute dialysis requirement	4 (6)
Estimated GFR (end follow up), mL/min/1.72 m^2^	82 (29.1)
Patients with End stage kidney disease	1(1.7)
Death (any cause)	3 (5)

**Table 2:** Comparison of demographics and clinical characteristics between patients who experienced a relapse and those who did not.

Characteristic	No Relapse *N* = 23	Relapse *N* = 24 (All fup)	*p*-Value
Age at start of symptoms (years)	51.65 (±17.3)	41.08 (±15.2)	0.03
Age at diagnosis (years)	51.73 (±17.3)	41.20 (±15.4)	0.03
Gender (males)	10 (0.4)	14 (0.6)	0.38
Menopause (yes)	3 (0.4)	6 (0.6)	0.34
Race (Caucasians)	19 (0.95)	24 (1)	0.45
Smoker	7 (0.3)	5 (0.5)	0.99
Current	4 (0.2)	3 (0.3)	
Ex-smoker	1(0.04)	0	
Never	2 (0.08)	2 (0.2)	
Body mass index	31 (±3.9)	23.1 (±3.5)	
History of diabetes mellitus	1(0.4)	1(0.04)	1
History of hypertension	9 (0.4)	7(0.3)	0.54
History of lung disease	2 (0.9)	0	0.23
Single kidney	0	1(0.4)	1
History of malignancy	2(0.9)	0	0.23

**Table 3:** Comparison of clinical and laboratory characteristics between relapsers and non-relapsers at diagnosis.

Characteristic	No Relapse *N* = 23	Relapse *N* = 24	*p*-Value
Duration of proteinuria prior to biopsy (weeks)	6.3 (±10.85)	11.5 (±33.3)	0.05
Serum creatinine at biopsy (mg/dL)	1.8 (±3.9)	0.9 (±0.4)	0.31
Serum total protein (g/dL)	4.9 (±0.85)	4.65 (±0.9)	0.27
Serum albumin (g/dL)	2.5 (±0.7)	2.45 (±0.7)	0.82
Serum cholesterol (mg/dL)	349.3 (±101.9)	382.5 (±99.9)	0.30
Triglycerides (mg/dL)	169.1 (±90.5)	220.05 (±91.4)	0.08
Estimated GFR (mL/min/1.73 m^2^)	78.4 (±31.9)	91.2 (±28.7)	0.16
24 h proteinuria biopsy (g)	7961(±4095)	7326 (±2704)	0.53
Urine analysis protein at biopsy			0.79
+	0	0	
++	3 (0.1)	2 (0.1)	
+++	1(0.5)	10 (0.4)	
>3+	9 (0.4)	11(0.5)	
Urine analysis hematuria at biopsy			0.58
+	9 (0.4)	8 (0.3)	
++	2 (0.04)	4 (0.2)	
+++	2 (0.04)	1(0.04)	
>3+	0	0	
Edema at biopsy			0.11
+	7 (0.3)	3 (0.1)	
++	5 (0.2)	8 (0.4)	
+++	6 (0.3)	2 (0.1)	
>3	3 (0.1)	7 (0.3)	

**Table 4:** Comparison of treatment and outcomes between patients with biopsy-proven MCD who experienced a disease relapse and those who did not after achievement of remission with immunosuppressive therapy.

Characteristic *N* (%) or Mean (SD)	No Relapse *N* = 23	Relapse *N* = 24	*p*-Value
ACE-Inhibitor	6 (0.3)	10(0.4)	0.35
AT-II	3 (0.1)	2(0.08)	0.66
Diuretics	3 (0.1)	8(0.33)	0.16
Other anti-hypertensives	9 (0.4)	3(0.125)	0.04
**Initial immunosuppressive therapy:** Number of patients treated with: Glucocorticoids alone Cyclosporine plus glucocorticoids Cyclophosphamide plus glucocorticoids Rituximab plus glucocorticoids	23 17 (73.9) 2 (8.7) 3 (13.0) 1 (0.4)	24 21 (88) 1 (4) 2 (8) 0	0.23 0.24 0.99 1
Patients with Remission	23 (1)	24(1)	0.48
Time to 1st Remission (days)	73.05 (±31.5)	72.8 (±58.1)	0.99
24 h proteinuria at 1st remission (mg/day)	1711(±1037)	668 (±266)	0.25
Serum creatinine at 1st remission (mg/dL)	0.9 (±0.3)	0.9 (±0.3)	0.91
Estimated GFR at 1st remission-(mL/min/1.73 m^2^)	84.5 (±25.4)	92.4 (±25.1)	0.32
Serum albumin at 1st remission (g/dL)	3.7 (±0.9)	3.6 (±0.7)	1
Serum cholesterol at 1st remission (mg/dL)	216.57 (±46.7)	310.6 (±134.5)	0.01
24 h proteinuria at end follow up, (mg)	289.22 (±271.1)	3631 (±1109.2)	0.38
Estimated GFR at end follow up, (mL/min/1.73 m^2^)	85.1 (±27.8)	92.6 (±23.9)	0.38
**Therapy for 1stRelapse** Number of patients treated with: Glucocorticoids alone Cyclosporine plus glucocorticoids Cyclophosphamide plus glucocorticoids	NA	16 (0.7) 3 (0.1) 4 (0.2)	
**Therapy for 2ndRelapse** Number of patients treated with: Glucocorticoids alone Cyclosporine plus glucocorticoids Cyclophosphamide plus glucocorticoids	NA	7 (0.5) 3 (0.2) 5 (0.3)	
**Therapy of 3rdrelapse** Number of patients treated with: Glucocorticoids alone Cyclosporine plus glucocorticoids Cyclophosphamide plus glucocorticoids	NA	3 (0.4) 2 (0.25) 3 (0.4)	
Patients with Acute Kidney Injury	4 (0.17)	0	0.04
Acute dialysis requirement	3 (0.1)	0	0.1
Patients with End Stage Kidney Disease	0	0	1
Death (any cause)	1(0.04)	1(0.04)	1

**Table 5:** Univariate and multivariate ordinal logistic regression analyses of patients with MCD who experienced a disease relapse and those who did not.

Variable	Unadjusted OR (95% CI)	Adjusted OR (95% CI)	*p*-Value
Age at onset (years)	0.95 (0.91–0.98)	0.91 (0.83–0.99)	0.035
Gender (males)	0.91 (0.29–2.82)	3.24 (0.47–22.21)	0.113
eGFR (mL/min/1.73 m^2^)	1.01 (0.99–1.03)	0.97 (0.93–1.02)	0.342
24-hrproteinuria (g)	0.90 (0.75–1.05)	0.77 (0.55–1.08)	0.065
Time to remission (months)	0.95 (0.82–1.09)	0.79 (0.59–1.06)	0.237
History of hypertension	0.77 (0.23–2.50)	2.28 (0.39–13.38)	0.341
**Initial immunosuppressive therapy**			
Cyclophosphamide + glucocorticoids	0.73 (0.11–4.70)	4.34 (0.29–64.83)	0.297
Cyclosporine + glucocorticoids	0.72 (0.05–9.74)	1.40 (0.07–27.60)	0.606
ACE inhibitors	1.32 (0.41–4.28)	6.87 (0.76–61.79)	0.185
ATII inhibitors	0.88 (0.17–4.36)	21.16 (0.88–507.81)	0.155

## Data Availability

Data are not available publicly due to ethical restrictions.
